# Mixed convolutional and long short-term memory network for the detection of lethal ventricular arrhythmia

**DOI:** 10.1371/journal.pone.0216756

**Published:** 2019-05-20

**Authors:** Artzai Picon, Unai Irusta, Aitor Álvarez-Gila, Elisabete Aramendi, Felipe Alonso-Atienza, Carlos Figuera, Unai Ayala, Estibaliz Garrote, Lars Wik, Jo Kramer-Johansen, Trygve Eftestøl

**Affiliations:** 1 Computer Vision Group, Tecnalia Research & Innovation, Derio, Spain; 2 Department of Communications Engineering, University of the Basque Country UPV/EHU, Bilbao, Spain; 3 Department of Signal Theory and Communications, Rey Juan Carlos University, Madrid, Spain; 4 Client Solutions Advanced Analytics, BBVA, Madrid, Spain; 5 Electronics and Computing Department, Mondragon Unibertsitatea, Faculty of Engineering (MU-ENG), Mondragón, Spain; 6 Norwegian National Advisory Unit on Prehospital Emergency Medicine (NAKOS), Oslo University Hospital and University of Oslo, Oslo, Norway; 7 Department of Electrical Engineering and Computer Science, University of Stavanger, Stavanger, Norway; St. Michael’s Hospital, CANADA

## Abstract

Early defibrillation by an automated external defibrillator (AED) is key for the survival of out-of-hospital cardiac arrest (OHCA) patients. ECG feature extraction and machine learning have been successfully used to detect ventricular fibrillation (VF) in AED shock decision algorithms. Recently, deep learning architectures based on 1D Convolutional Neural Networks (CNN) have been proposed for this task. This study introduces a deep learning architecture based on 1D-CNN layers and a Long Short-Term Memory (LSTM) network for the detection of VF. Two datasets were used, one from public repositories of Holter recordings captured at the onset of the arrhythmia, and a second from OHCA patients obtained minutes after the onset of the arrest. Data was partitioned patient-wise into training (80%) to design the classifiers, and test (20%) to report the results. The proposed architecture was compared to 1D-CNN only deep learners, and to a classical approach based on VF-detection features and a support vector machine (SVM) classifier. The algorithms were evaluated in terms of balanced accuracy (BAC), the unweighted mean of the sensitivity (Se) and specificity (Sp). The BAC, Se, and Sp of the architecture for 4-s ECG segments was 99.3%, 99.7%, and 98.9% for the public data, and 98.0%, 99.2%, and 96.7% for OHCA data. The proposed architecture outperformed all other classifiers by at least 0.3-points in BAC in the public data, and by 2.2-points in the OHCA data. The architecture met the 95% Sp and 90% Se requirements of the American Heart Association in both datasets for segment lengths as short as 3-s. This is, to the best of our knowledge, the most accurate VF detection algorithm to date, especially on OHCA data, and it would enable an accurate shock no shock diagnosis in a very short time.

## Introduction

Worldwide out-of-hospital cardiac arrest (OHCA) has an average incidence of 55 cases per 100 000 person-year [1], and constitutes one of the leading causes of death in the industrialized world. Lethal ventricular arrhythmia such as pulseless ventricular tachycardia (VT) and ventricular fibrillation (VF) are the most frequent trigger of cardiac arrest [2]. In fact, ventricular arrhythmia are observed as initial rhythm in up to 75% of cases if recorded right after the collapse by an on-site automated external defibrillator (AED) [3]. An electric defibrillation shock is the only effective therapy to revert VF/VT and restore a normal rhythm. AEDs are simple devices designed for untrained lay people, and thus incorporate a shock/no-shock decision algorithm based on the analysis of the patient’s electrocardiogram (ECG) [4]. When the algorithm detects a shockable rhythm (VF/VT) the device delivers a defibrillation shock to restore a perfusing rhythm, otherwise the AED recommends continuation of cardiopulmonary resuscitation (CPR). The American Heart Association (AHA) established the minimum accuracy requirements for shock decision algorithms to ensure a safe use of the device, and that the adequate therapy is administered [5]. Shockable rhythms should be detected with a sensitivity (positive class) above 90%. The specificity for nonshockable rhythms (negative class) should be above 99% in case of normal sinus rhythms, and above 95% for other arrhythmia like atrial fibrillation, supraventricular tachycardia, ideoventricular rhythms, heart blocks, or bradycardia.

The development of automatic shock decision algorithms has been an active field of research for decades. The ECG of lethal ventricular arrhythmia present some distinctive characteristics such as more waveform irregularity, the absence of narrow or wide QRS complexes (the ECG waveform deflections associated to ventricular contraction), smaller bandwidths, and higher ventricular rates. Consequently, initial efforts focused on the development of ECG features to identify VF, including features to quantify amplitudes [6, 7], waveform correlation [8], heart rate [9, 10], slope [11, 12], spectral [13–15] and time-frequency characteristics [16–18], or the complexity of the VF waveform [19–21]. Many of these features have been systematically reviewed and compared [22–25] using data from public ECG repositories available through the physionet initiative [26]. Current findings, however, support the use of ECG from OHCA patients gathered by defibrillators during treatment because the ECGs may be very different both for shockable and nonshockable rhythms [27]. Recently, efforts have focused on efficiently combining ECG features for VF detection through state of the art machine learning techniques like support vector machines (SVM) or random forests [27–33]. These latter studies explore the limits of the accuracy attainable by a feature extraction approach using both data from public repositories [28, 29, 31, 32], and from OHCA cases [27, 30].

Machine learning paradigms based on feature extraction and feature selection strongly depend on the design of the features. An alternative approach is to exploit all available knowledge from the signals and to let the machine learning algorithm learn and select those features. This is the rationale behind deep learning, and in particular convolutional neural networks (CNN), and their one dimensional counterparts 1D-CNN, which have been recently introduced for patient specific heartbeat classification [34], arrhythmia classification [35], detection of myocardial infaction [36], detection of ectopic beats [37], and the detection arrhythmia [35] including atrial fibrillation [38] and VF [39]. ECG-based arrhythmia classification using CNNs has been shown to be more accurate than that of clinicians [40, 41], and 1-D CNNs have outperformed classical feature extraction-classification paradigms in ECG beat classification [42]. When there are time dependencies and variable segment lengths in the data Recurrent Neural Networks (RNN) are an efficient deep learning approach [43], and in particular some of its variants like Long Short-Term Memory (LSTM) networks [44, 45]. In fact, LSTMs have been proven accurate in diagnostics [46], but also for the detection of anomalous heartbeat contractions using the ECG [47].

This study introduces a deep learning approach that combines a CNN and an LSTM network for the detection of lethal ventricular arrhythmia using data from both public ECG repositories and from defibrillators used to treat OHCA patients. The paper is organized as follows. The methodology is described in materials and methods, including a description of the data, the mixed CNN+LSTM architecture proposed in this paper, and a classical machine learning approach using SVMs used for baseline comparisons. The results section compares the baseline classifier and 3 deep learning architectures based only on CNNs to our mixed CNN+LSTM architecture, analyzes the influence of shortening the analysis segment length, and evaluates the classification features learned by our deep learner. In the discussion the results are put in the context of previous findings on the detection of lethal ventricular arrhythmia, with emphasis on the importance of using OHCA data in these studies.

## Materials and methods

### Data preparation

The data used in this study come from a previous study on classical machine learning approaches for the detection of ventricular arrhythmia [27]. What follows is an abridged description of the data, the ECG preprocessing, and data partitioning for training and testing. For further details consult [27].

#### Datasets

**Fig 1:**
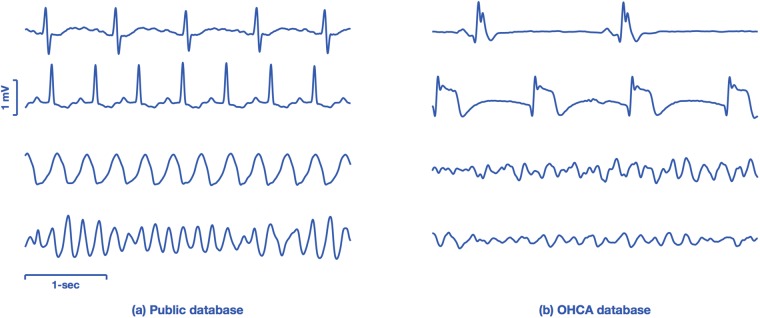
ECG samples from the public and OHCA datasets. The examples on the left panel show ECGs from public databases, ranging from normal (top) to VT and VF in the bottom most examples. The ECGs on the right come from the OHCA dataset, and the two topmost cases are organized rhythms (without pulse) but with very aberrant QRS complexes. The two examples in the bottom are VF observed minutes after the arrest occurred, and have smaller amplitudes and fibrillation frequencies than those observed in the public databases (bottom-left).

The data comprise two datasets, one originated from public repositories of arrhythmia (public database), and a second dataset from OHCA data recorded by monitor-defibrillators during treatment (OHCA dataset). Fig 1 shows four characteristic examples of 4-s ECG segments from both databases. The examples illustrate the differences between the ECGs found in public databases, which come from continuously monitored patients (Holter records), and the rhythms seen by a monitor-defibrillator attached to patient several minutes after the onset of cardiac arrest.

The public dataset was formed using three databases. It includes the complete record set from the physionet MIT-BIH Malignant Ventricular Arrhythmia Database (VFDB) and the Creighton University Ventricular Tachyarrhythmia Database (CUDB), and ten episodes (8201-8210) from the series 1 American Heart Association ECG Database (AHADB) [26]. When more than one ECG channel were available (VFDB and AHADB) only the first channel was extracted to avoid redundancies, as done in recent studies on this topic [27, 39, 48]. The records included arrhythmia annotations in the Physionet’s scheme, and all databases included recordings with lethal ventricular arrhythmia from the onset of the arrhythmia. The sample rate of all databases was *f*_*s*_ = 250 Hz.

The OHCA database originated in a multicenter OHCA study conducted in Akershus (Norway), Stockholm (Sweden), and London (UK) between 2002-2004 [49, 50]. The original studies were approved by the regional ethics committees and complied with the Helsinki declaration. Rhythms were annotated by expert clinicians into five classes [30]: VF, VT, asystole (AS), and organized rhythms with pulse (Pulsed Rhythms, PR) and without pulse (Pulseless Electrical Activity, PEA). ECG segments of 10 s duration and without CPR artifacts were included in the dataset. The ECG was acquired through the defibrillation pads with a resolution of 1.031 *μ*V per least significant bit and a sampling frequency of 500 Hz. The ECG was resampled to *f*_*s*_ = 250 Hz.

#### ECG preprocessing and data labeling

The ECG was filtered in all datasets following a typical preprocessing introduced in [24] which consists of: mean subtraction, a moving average filter, and bandpass filtering in the typical 1 − 30 Hz AED monitoring bandwidth [51, 52]. This is the typical preprocessing found in AEDs, although the cutoff frequencies may differ across AED models. Preprocessing removes low frequency noise (movement, respiration, …) and high frequency noise (power line interference) that may confound the classification algorithms.

Ground truth rhythm labels were reviewed by consensus among two experienced biomedical engineers specialized in cardiac arrest rhythms. The process included: identification of device saturation intervals, annotation of noise intervals, identification of very low amplitude VF (peak amplitude < 200 μV), and slow VT (rate under 150 bpm), and identification of asystole defined as rhythms with very low rates (under 12 bpm) and/or peak-to-peak amplitude (< 100 μV).

The ECG signals were then divided into non-overlapping segments of 2, 3, 4, and 8-s, to analyze the accuracy of the shock decision methods in terms of the ECG segment length. Segments with noise, low-quality signal or artifacts were discarded. Following the AHA statement, only segments with a unique rhythm label were considered. Slow VT and fine VF were discarded because the benefits of defibrillation are unclear for these rhythms [5]. Consequently, they cannot be unequivocally labeled as shockable or nonshockable. Finally, segments labeled as AS were also discarded [27, 29], because normally AS is identified before the shock decision algorithm using simple thresholding based on the amplitude/power of the ECG segment [11, 53]. In total 86.5% of available data was used from the public dataset, 96.3% of the AHADB, 82.6% of the VFDB and 85.2% of the CUDB. This was the subset used by Figuera et al [27], and the curated public datasets with noise and unusable signal annotations are available from there.

Table 1 shows the final datasets for the different segment lengths grouped as shockable and nonshockable. Shockable rhythms include VF, VT, and ventricular flutter, and nonshockable rhythms include organized rhythms (PEA/PR) from the OHCA database, and normal sinus rhythm, supraventricular tachycardia, atrial fibrillation, heart blocks, or ectopic ventricular activity from public databases.

**Table 1:** Dataset used for shock decision algorithms for different segment lengths grouped by shockable (Sh) and nonshockable (NSh) segments.

Database	2-s segments		3-s segments		4-s segments		8-s segments	
	Sh	NSh	Sh	NSh	Sh	NSh	Sh	NSh
**Public**	**7407**	**29365**	**4842**	**19450**	**3578**	**14495**	**1696**	**7086**
vfdb	3318	15757	2149	10427	1586	7761	746	3780
cudb	1510	6075	981	4015	716	2986	323	1446
ahadb	2579	7533	1712	5008	1276	3748	627	1860
**OHCA**	**1700**	**3235**	**1020**	**1941**	**680**	**1294**	**340**	**647**

#### Data partitioning

Data was partitioned patient-wise into two sets, 80% of patients to train the classification algorithms and 20% to report the performance of the algorithms. The split was done randomly on each of the four databases separately (VFDB, CUDB, AHADB, and OHCA), and it is the same split used in the study that originated the datasets [27]. Then, all four training sets were merged into a single dataset to train the models. The results were evaluated independently for two test sets, the public test set obtained merging the three test set from the public databases (VFDB, CUDB, AHADB), and the OHCA test set.

### Deep learning architecture

#### Proposed architecture

**Fig 2:**
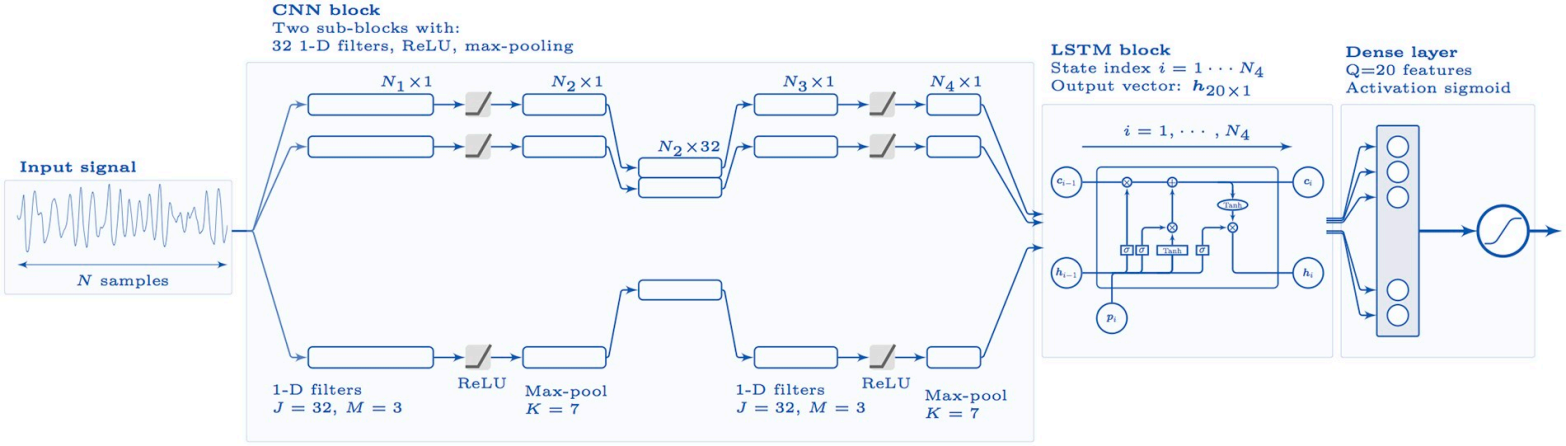
Architecture of the deep learning network. The architecture has three blocks: a convolutional block (CNN), a recursive block (LSTM), and a final decision stage based on a neural network.

Fig 2 shows the architecture of our deep learning classifier, which comprises three stages: a convolutional block with two 1D-CNN stages, an LSTM network, and a classification stage. The convolutional block extracts the high level descriptors from the ECG signal, and creates a feature map representing the ECG signal that maintains its temporal order. The long and short time relations in the feature map are integrated by the LSTM network that outputs *Q* features that are used by a dense neural network that outputs *p*_Sh_ ∈ [0, 1], the likelihood that the ECG corresponds to a shockable rhythm. *p*_Sh_ was used for the shock/no-shock decision.

**1D-CNN**. The convolutional block is composed of two convolutional sub-blocks with a similar architecture. Each sub-block starts with a convolutional layer of *J* = 32 filters of size *M* = 3, followed by a rectified linear unit (ReLU) as nonlinear activation function. The ReLU unit is followed by a max-pooling unit of size *K* = 7 to reduce the dimensionality. The two convolutional sublocks result in a time-compressed signal rich enough for feature extraction.

Mathematically the architecture (in the forward direction) of the network is explained as follows. Let us represent the input signal segment as a column vector of *N* ordered samples by: ***s*** = {*s*_*i*_}_*i* = 1, …, *N*_ = (*s*_1_, *s*_2_, …, *s*_*N*_)^*T*^, where *N* = *L* ⋅ *f*_*s*_ for a segment of length *L* seconds. A convolutional layer with *j* = 1, …*J* filters of size *M* and ReLU activation function *f*(⋅), has the following outputs:

$$c_{i}^{j}=f(b_{j}+\sum_{m=1}^{M}\omega_{m}^{j}\cdot s_{i+m-1}^{j})\;\text{with}\;f\left(\cdot \right)=\text{max}\left\{0,\cdot \right\},$$ (1)

where $\omega_{m}^{j}$ is the *m*-th coefficient in the *j*-th filter of the network, and ***s***^*j*^ its input signal. At the max-pooling stage each output signal is down-sampled by a factor of *K*, the pool size, by applying maximum filter to non-overlapping *K* sample segments:

$$p_{i}^{j}=\text{max}\{c_{k}^{j}{\}}_{k=(i-1)\cdot K+1,\ldots ,i\cdot K}$$ (2)

The temporal length, *N*_*ℓ*_, at each of the four phases (*ℓ* = 1, 2, 3, 4) of the two-stage CNN network is given by (see Fig 2):

$$N_{\ell }=\text{floor}(\frac{N_{\ell -1}+2p-M}{s})+1$$ (3)

where *N*_0_ = *N* is the length of the input signal, *p* = 0 is the padding, *M* is the kernel size (filter size, max-pooling size), and *s* is the stride which is *s* = 1 for the convolutional layers and *s* = *K* for the max-pooling layers. So for an 8-s segment the successive lengths would be (*M* = 3 and *K* = 7):

$$N=8\cdot f_{s}=2000\Rightarrow N_{1}=1998\Rightarrow N_{2}=285\Rightarrow N_{3}=283\Rightarrow N_{4}=40$$ (4)

and the input to the LSTM network would have a temporal index of *i* = 1, …, 40.

**LSTM**. The output of the convolutional network feeds the LSTM network in which the vector with the current state (value) of the input signal is $p_{i}={\left(p_{i}^{1},\ldots ,p_{i}^{J}\right)}^{T}$ with *i* = 1, …, *N*_4_. The LSTM network maps the current output signal state $h_{i}={\left(h_{i}^{1},\ldots ,h_{i}^{Q}\right)}^{T}$ to the previous states of the input and output by means of a network of consecutive recurrent cells that consist of various gates with sigmoid activation functions *σ*(⋅).

The equations for the LSTM network in the forward direction are [43–45]:

$$f_{i}=\sigma \left(W_{f}\;p_{i}+R_{f}\;h_{i-1}+b_{f}\right)$$ (5)

$$i_{i}=\sigma \left(W_{i}\;p_{i}+R_{i}\;h_{i-1}+b_{i}\right)$$ (6)

$$o_{i}=\sigma \left(W_{o}\;p_{i}+R_{o}\;h_{i-1}+b_{o}\right)$$ (7)

$$c_{i}=f_{i}⊙c_{i-1}+i_{i}⊙\text{tanh}\left(W_{c}\;p_{i}+R_{c}\;h_{i-1}+b_{c}\right)$$ (8)

$$h_{i}=o_{i}⊙\text{tanh}\left(c_{i}\right)$$ (9)

where the $W_{Q\times J}$ and $R_{Q\times Q}$ are the input and recurrence weight matrices, respectively, and ***b***_*Q*×1_ are the bias vectors. The number of output features of our network is *Q* = 20. The ***f***_*i*_, ***i***_*i*_, ***o***_*i*_, ***c***_*i*_ vectors correspond to the forget, input, output, and state vectors of the *i*-th cell in the LSTM architecture, and ⊙ denotes Hadamard or element-wise product.

**Fully connected layer**: The 20 feature output vector of the LSTM network, ***h***_*i*_ for *i* = *N*_4_, is densely connected to a classical neural network with one sigmoid output layer that outputs *p*_Sh_. Finally, *p*_Sh_ is compared to a threshold to output a binary shock/no-shock decision. The decision threshold that maximized the balanced accuracy of the training set was selected once the model was optimized.

At the training stage, the network was optimized to minimize the binary cross-entropy loss function (*L*), defined as:

$$L=\sum_{i}\eta_{i}[y_{i}\ln\left(p_{{\text{Sh}}_{i}}\right)+\left(1-y_{i}\right)\ln\left(1-p_{{\text{Sh}}_{i}}\right)]$$ (10)

where *η*_*i*_ is the weight of instance *i*. Weights were assigned to address class imbalance and to give equal weight to shockable and non-shockable classes during training. Optimization was done using Stochastic Gradient Descent with Nesterov acceleration, a learning rate of 10^−3^, a learning rate decay of 10^−6^, and a momentum of 0.9. These are recommneded/typical values used in Stochastic Gradient Descent from the specialized literature on the topic [54, 55]. One of the advantages of our design is that the three blocks of the network are optimized end-to-end to simultaneously extract the high level feature description of the signal (convolutional block), its temporal relationships (recurrent network block), and the arrhythmia classification (classification block).

#### CNN only networks

A number of CNN only networks have been previously introduced for heartbeat [34, 56] and arrhythmia classification [39]. These approaches proved that it is possible to obtain high level descriptors of the ECG using only 1-D convolutional blocks, although they differed on the characteristics of the networks such as depth, filter lengths or max-pooling size. We have implemented three representative examples (see Table 2) and compared their performance to our architecture, in which an additional LSTM block is added to better characterize the temporal relations of the arrhythmia.

**Table 2:** Architectures of the CNN only models used for comparison.

CNN	Layers	Filters	Filter size	Max-pool size	Dense layers
Kiranyaz et al [34]	2	32, 16	15, 15	6	1
Zubair et al [56]	3	32, 16, 8	5, 5, 5	2	1
Acharya et al [39]	4	3, 5, 10, 10	5, 5, 5, 4	2	2

All the experiments on the deep learning models were done using a Tesla V100-DGXS-16Gb GPU, and were based on the Keras framework [57] with Tensorflow backend [58]. The CNN only and the proposed CNN-LSTM architectures were trained using 600 epochs, at each epoch all training data were fed to the models in batches of 256.

#### Baseline learner

A SVM classifier with Gaussian kernel [59] was optimized to obtain a baseline performance for the shock decision algorithm. SVMs for the detection of VF have been demonstrated before on data from public databases [27, 29, 31], and on OHCA studies [27]. In the feature extraction phase 30 classical VF detection features were computed, comprising all the analysis domains of the ECG. The code for feature extraction is available through [27]. Two configurations of the SVM were tested, with all 30 features, and with the top 8 features as selected in [27] using ensemble methods. These will be termed SVM_all_ and SVM_sel_ in what follows.

Our dataset can thus be represented by a set of instance-label pairs $\{\left(x_{1},y_{1}\right),...,\left(x_{n},y_{n}\right)\}\in R^{K}\times \left\{\pm 1\right\}$, where *y*_*i*_ = 1 for shockable and *y*_*i*_ = −1 for nonshockable rhythms, and the vector ***x***_*i*_ contains the values of the *K* features for ECG segment *i*. The decision function of the SVM is found by solving the following maximization problem [59]:

$$\max_{\alpha_{i}}\{\sum_{i=1}^{n}\alpha_{i}-\frac{1}{2}\sum_{i,j=1}^{n}\alpha_{i}\alpha_{j}y_{i}y_{j}\exp\left(-\gamma {∥x_{i}-x_{j}∥}^{2}\right)\}$$ (11)

$$\text{s}.\text{t}.:\;0\leq \alpha_{i}\leq C\;\forall i,\;\text{and}\;\sum_{i=1}^{n}\alpha_{i}y_{i}=0$$ (12)

After solving the maximization problem, the vectors that have non-zero Lagrange multipliers (*α*_*i*_) are called support vectors. The SVM decision function depends only on the support vectors, and can be written as:

$$f\left(x\right)=\text{sign}[\sum_{i=1}^{N_{s}}\alpha_{i}y_{i}\exp(-\gamma ∥x-x_{i}{∥}^{2})+b]$$ (13)

where the *N*_*s*_ support vectors and the threshold *b* are determined in the optimization phase. A rhythm will be classified as shockable for *f*(***x***) = 1, or nonshockable for *f*(***x***) = −1.

The gaussian kernel SVM has two optimization hyper-parameters, *C* is the soft margin parameter and *γ* the width of the gaussian kernel. Bayesian optimization was used to determine the optimal hyper-parameters of the SVM in the training data, and the hyperparameter search region was bound to 10^−3^ ≤ *C* ≤ 2 ⋅ 10^2^ and 10^−3^ ≤ *γ* ≤ 2 ⋅ 10^2^. Class imbalance was addressed by weighting the instances in the training data to match the total weight of the shockable and nonshockable classes. Finally, at most 0.5% of instances were discarded as outliers during training, this number is roughly equivalent to applying the 3-*σ* rule for a normal distribution [60].

### Performance metrics and evaluation

The performance of the classification algorithms was evaluated using the test sets. Shock decision algorithms are binary classifiers, so results are reported in terms of sensitivity (Se) and specificity (Sp), positive and negative predictive values (PPV and NPV), and two measures of global accuracy, total accuracy (Acc) and balanced accuracy (BAC), which is defined as:

$$\text{BAC}=\frac{1}{2}\left(\text{Se}+\text{Sp}\right).$$

The AHA statement gives a similar importance to the detection of shockable (Se) and nonshockable (Sp) rhythms, by setting the minimum performance goals for Se and Sp at 90% and 95%, respectively. Consequently, we ranked our solutions by their BAC, which is simply the unweighted mean of sensitivities for a two-class problem. BAC is less sensitive to class imbalance than total accuracy [30]. Since our data is strongly imbalanced (roughly by a proportion of 4/1 in all datasets), we assigned weights as inverses of the class prevalences to the training instances on the optimization phase of all the classifiers. The McNemar test was used to test the null hypothesis that the accuracy of our model was greater than that of the other models at the 95% confidence interval, that is a *p* < .05 was considered significant [61].

The discriminating power of the features learned by our CNN+LSTM architecture was evaluated using a Receiver Operating Curve (ROC) analysis in the test set. In our architecture, those features are the 20 components of the output signal state at time *i* = *N*_4_, $h_{N_{4}}\in R^{Q}$ of the LSTM block. We will denote those features by lstm_*j*_ for *j* = 1, …20. The CNN+LSTM model was trained using the training data, and the trained model was used to compute the LSTM features in the training and test sets. The value of the LSTM features output by the model for the test set was used for the ROC curve analysis. The features were ranked using the Area Under the Curve (AUC) of the ROC analysis.

## Results

### Performance of the classification algorithms

Table 3 shows the performance metrics for 4-s segments in the test dataset, both for the public and OHCA databases. As shown in the table the mixed deep learning architecture proposed in this study presents the best performance, and the McNemar test showed that the accuracy of our model was significantly better than that of the rest of the classifiers. The differences between our solution and the best CNN and SVM solutions are only marginal for the public dataset, but very large for the OHCA database with differences in BAC and Acc of over 2.2-points and 1.4-points, respectively. A 1.4-point increase in Acc from a baseline of 96.1% (for SVM_sel_) means that over 35% of the errors are now corrected.

**Table 3:** Performance metrics for 4-s segments. The the p-values are for the pairwise comparison of the accuracy of the proposed model to each of the other classifiers. The proposed network had a highest accuracy and the results were significant at the 95% confidence level (indicated by a^†^).

Method	Public datasets				OHCA datasets			
	Se/Sp	BAC/Acc	PPV/NPV	p-val	Se/Sp	BAC/Acc	PPV/NPV	p-val
**Proposed network**	**99.7/98.9**	**99.3/99.1**	**96.3/99.9**	-	**99.2**/96.7	**98.0/97.5**	93.6/**99.6**	-
**Deep learners**								
Kiranyaz et al [34]	99.3/98.4	98.9/98.6	94.6/99.8	< .05^†^	97.7/93.8	95.8/95.1	88.4/98.8	< .05^†^
Zubair et al [56]	95.6/96.2	95.9/96.1	87.5/98.7	< .05^†^	93.9/89.4	91.7/90.9	81.0/96.8	< .05^†^
Acharya et al [39]	98.4/98.1	98.2/98.2	93.6/99.5	< .05^†^	94.7/91.6	93.2/92.6	84.5/97.3	< .05^†^
**Baseline SVM**								
SVM_all_	98.5/98.3	98.4/98.4	94.3/99.6	< .05^†^	91.7/96.0	93.9/94.6	91.7/96.0	< .05^†^
SVM_sel_	99.2/98.7	99.0/98.8	95.5/99.8	< .05^†^	93.9/**97.1**	95.5/96.1	**93.9**/97.1	< .05^†^

For 4-s segments, the time needed to train our model (CNN+LSTM) in our high-end architecture was 1 hour and 3 min. The CNN only models were much quicker to train, with training times below 5 min. The time needed to classify a test sample (without parallelization) using the trained models was of 245 ms for our model, and below 14 ms for the CNN only models. All the deep learning models could be easily implemented and used in real time in low end hardware like the Field Programmable Gate Arrays (FPGA) and low end micro-processors used in AEDs [62].

### Dependence with the length of the analysis segment

**Fig 3:**
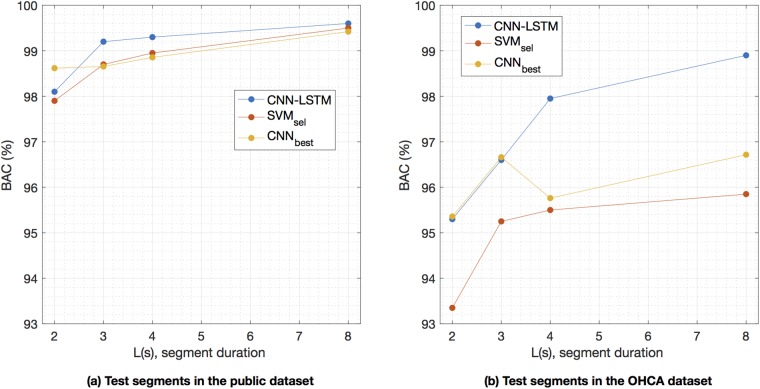
Performance for different segment lengths. Performance of the proposed network and the best baseline SVM and CNN only solution [34] for different segment lengths. The performance is worse for the OHCA database. The proposed solution outperforms the baseline SVM in both databases for all segment lengths (*p* < .05), and the best CNN solution (*p* < .05) except for short segment lengths.

The BAC of the algorithm as the analysis segment is shortened is shown in Fig 3. The algorithm’s accuracy is preserved for ECG analysis segments longer than 3-s, with a BAC above 99% in the public database and above 97% for the OHCA database. Although the accuracy is still good for 2—segments, the performance of the shock decision algorithms degrades. As shown in the figure, the proposed architecture outperforms the baseline SVM classifier (*p* < .05 for the McNemar test for all segment lengths) specially in the OHCA database. And it performs better than the best CNN only solution (*p* < .05) for longer segment lengths, and similarly for small segment lengths (see S1 Fig for the comparison with the rest of the CNN models).

**Fig 4:**
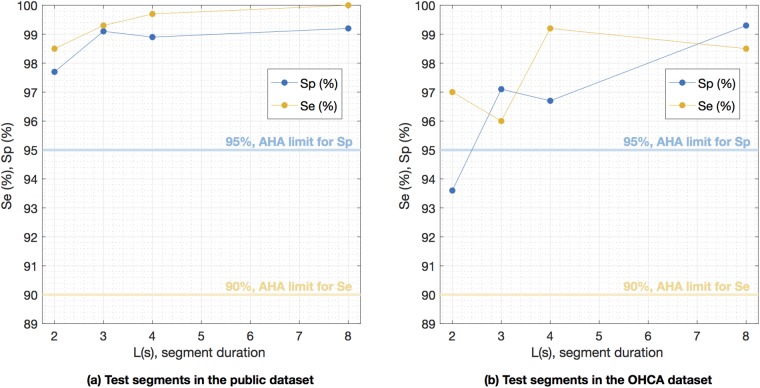
Sensitivity and specificity for different segment lengths. Sensitivity and specificity of the proposed deep learning network for different segment lengths for the public and OHCA databases. The algorithm meets AHA’s minimum 90% sensitivity for all segment lengths, and the 95% specificity for segment lengths longer than 2 s.

Our results show that a deep learning architecture can accurately detect shockable rhythms with very short ECG segments, even on data from OHCA patients. Furthermore, the algorithm is AHA compliant for all segment lengths and datasets (except 2-s segments on OHCA data), as shown in Fig 4. The sensitivity and specificity were always above their respective minimum AHA goals of 90% and 95%.

### Learned and designed features

One of the salient characteristics of deep-learning architectures is their ability to automatically learn the discrimination features. Table 4 shows the AUCs of the lstm_*j*_ features on the test dataset for 4-s segments. Features are ordered using the AUC for the complete dataset (public+OHCA) because the features presented a mixed ranking when evaluated on the public and OHCA datasets separately. Six features had global AUCs above 95%, and are therefore very good individual shock/no-shock decision features. In most cases the AUCs for the public database were larger than for the OHCA database, which confirms that accurate shock/no-shock decisions are more difficult for OHCA rhythms. However the network was also able to learn several features that are better suited for OHCA data, such as lstm_9_ or lstm_1_.

**Table 4:** AUCs on the test set of the LSTM features of proposed architecture (see Fig 2). The model was trained in the training set for 4-s ECG segments, and this model was used to compute the LSTM features in the test set. The features are ordered by descending AUCs on the complete test set (public+OHCA).

Feature	Dataset			Feature	Dataset		
	Complete	Public	OHCA		Complete	Public	OHCA
lstm_4_	99.12	99.34	96.96	lstm_5_	91.19	91.10	92.57
lstm_6_	98.81	99.19	95.13	lstm_3_	85.91	85.44	90.24
lstm_19_	98.53	99.34	94.99	lstm_8_	85.84	85.93	88.43
lstm_14_	96.64	96.72	97.33	lstm_10_	83.15	84.35	82.93
lstm_11_	95.39	95.62	90.95	lstm_17_	80.19	81.40	72.90
lstm_15_	95.29	97.13	78.30	lstm_9_	79.80	78.39	88.32
lstm_16_	94.47	94.89	92.24	lstm_1_	78.91	76.87	90.97
lstm_18_	94.19	94.35	92.83	lstm_12_	75.47	75.61	83.91
lstm_13_	92.49	93.19	88.70	lstm_2_	66.97	67.84	56.51
lstm_17_	91.52	81.40	72.90	lstm_20_	56.80	57.00	50.87

**Fig 5:**
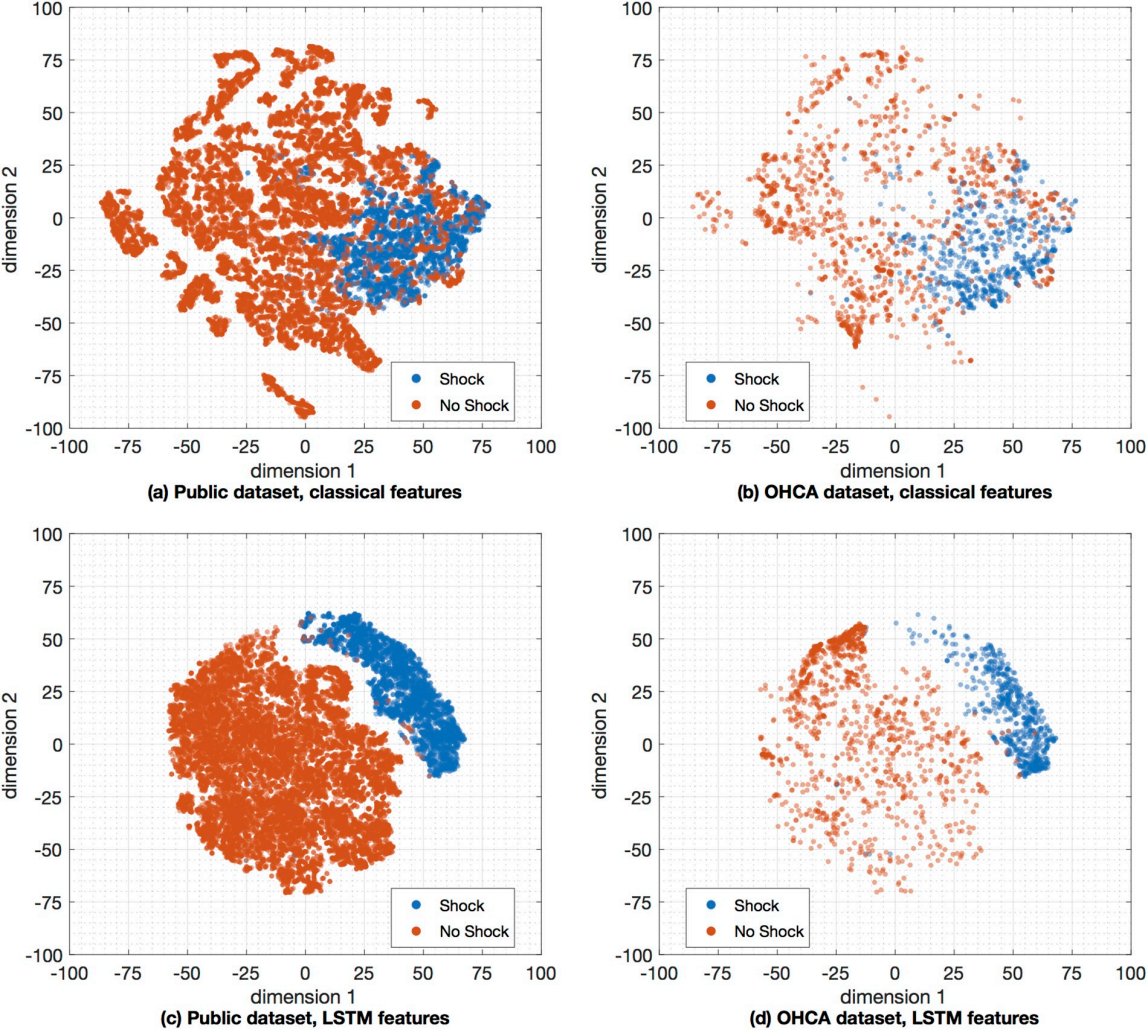
Visualization by t-SNE of the separability for the learned and classical VF-detection features. Two dimensional map visualization using t-SNE with the Barnes-Hut algorithm based on 30 classical VF-detection features [27] (top), and the 20 LSTM features (bottom). All experiments were done for the 4-s segments, and the visualization is separated for the public (left) and OHCA (right) datasets.

We then applied dimensionality reduction techniques to visually assess and compare the classical VF-detection features to the features learned by our deep learning architecture. We choose the t-Distributed Stochastic Neighbor Embedding (t-SNE) dimensionality reduction technique [63, 64], which is a nonparametric nonlinear technique well suited for 2-D representations that has been shown to outperform many typical dimensionality reduction techniques in problems ranging from hand-digit recognition to genomic data clustering [63, 65]. In our configuration we reduced 30 classical features [27] and 20 LSTM features computed using 4—segments to a 2-D map suitable for visualization using scatterplots. The results for the public and OHCA datasets are shown in Fig 5, respectively. It is visually apparent that LSTM features produce a better and more separable clustering than the classical features. In fact, the Davies-Boudin index to measure the separability of the clusters [66] was significantly lower for the learned features on 1000 bootstrap replicas of the experiment. The values mean (standard deviation) values were 1.073 (.006) for the LSTM features and 1.472 (.013) for the hand-crafted features (*p* < .05). These numbers confirm that the LSTM features produced a better clustering of the two classes than the classical VF-detection features.

**Fig 6:**
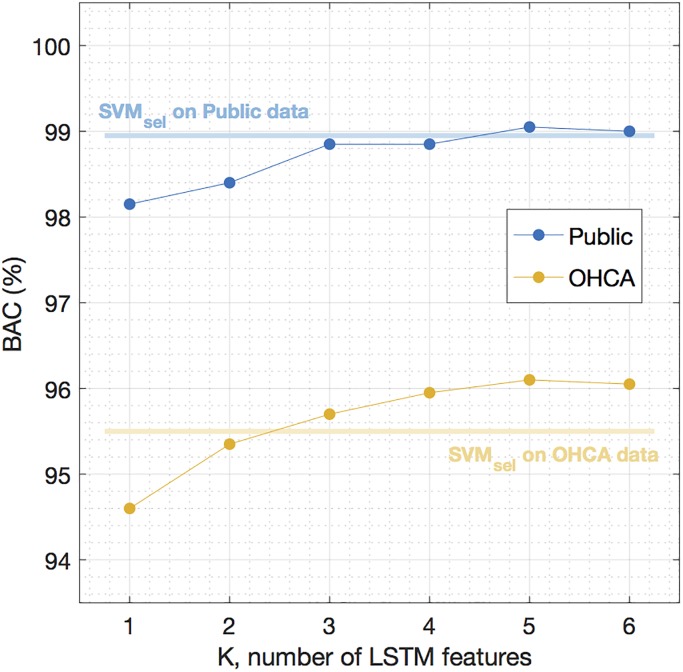
LSTM features used in an SVM classifier. Performance of an SVM classifier based on LSTM features, compared to the best baseline classifier using classical features, SVM_sel_.

Finally, we redesigned the classical SVM learner but fed it with the LSTM features instead, in line with a recent proposal that uses learned features from a 1-D CNN to detect VF [48]. The objective was to show if, for a classical machine learning approach based on SVM, the accuracy improved using the LSTM features instead of the classical VF-features. And if so, to determine the minimum number of LSTM features needed to improve those classification results. All the experiments were done using the features obtained from the 4-s segments, and the SVM classifiers with LSTM features were optimized and evaluated using the same procedures used for the baseline SVM. First, on the training data a simple linear discriminant analysis classifier was used to select the best combination of LSTM features for all the possible feature sets of *K* = 1, 2, …20 features. Then, for each value of *K* the SVM classifier was optimized on the training data, and its performance was evaluated in the test set. The results are shown in Fig 6, compared to the best baseline SVM learner based on classical VF-features. The SVM based on LSTM features outperformed the SVM based on classical features for as few as *K* = 4 LSTM features, and using the LSTM features improved the accuracy particularly for the most challenging data, the OHCA dataset.

### Sources of classification errors

**Fig 7:**
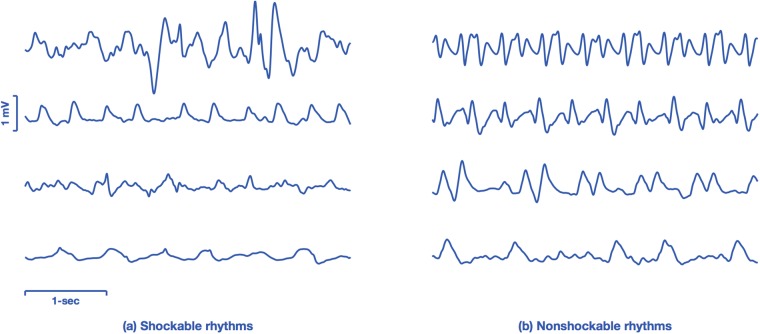
Examples of classification errors from the OHCA set. Examples of 4-s segments corresponding to borderline rhythms that were misclassified by the deep learning architecture. Segments corresponding to shockable rhythms are shown in the left, and segments corresponding to nonshockable rhythms in the right.

Fig 7 shows eight 4-s segments from the OHCA dataset incorrectly classified by our deep learning architecture. The examples shown in the figure illustrate the difficulty of classifying certain borderline OHCA rhythms. The four examples on the left (Fig 7(a)) show rhythms labeled as shockable that were misclassified as nonshockable by our algorithm. The first missed shockable rhythm presents several bursts of high amplitude activity, which result in large slopes in the ECG that are sometimes interpreted as QR segments, although no distinct QRS complexes are visible. In the second example the rhythm is ventricular and regular, but the ventricular rate is around 150 bpm, a threshold used frequently to separate intermediate VT from shockable fast VT. Finally the two bottom-most examples correspond to the late stages of VF in which the amplitude and fibrillation frequencies are lower, and the rhythm starts to degenerate into asystole.

The four examples on the right (Fig 7(b)) show rhythms labeled as nonshockable that were misclassified as shockable by our algorithm. The two topmost examples of missed nonshockable rhythms correspond to two supraventricular rhythms but with very wide QRS complexes and unstable heart rate, which result in a more irregular ECG than normally seen for nonshockable rhythms. The bottom most examples correspond to PEA cases with no supraventricular activity, but low rates. In the bottom most example the ventricular rate is very slow (around 60 bpm), but there is a high frequency quivering between ventricular contractions that produces a very irregular ECG.

## Discussion

This is, to the best of our knowledge, the first study that uses OHCA data to develop and evaluate a deep learning architecture for the detection of shockable arrhythmia during cardiac arrest. In addition, we have for the first time analyzed the influence of the segment length on the performance of the deep learning classifier, and we have done an in-depth analysis of the features learned by our architecture when compared to the classical features used for the detection of VF over the past two decades. When compared to other deep learning approaches and classical machine learning approaches our architecture showed the best accuracy for the shock/no-shock decisions, specially of OHCA data. Our solution would therefore increase the security of AED algorithms by avoiding inappropriate shocks, and would ensure shocks are delivered when the patient is in VF. Furthermore, the algorithm fits within the computational and memory constraints of the low-end hardware of an AED. Therefore it could be implemented simply by changing the shock decision algorithm of the AED, i.e. a low cost software change.

Shock advice algorithms in current AED are safe, they are tested following the AHA framework [5] and IEC standards. Testing of those algorithms is done using AHA compliant vendor proprietary rhythm libraries, and the algorithms meet the AHA requirements for Se/Sp. In fact, many of those algorithms have been recently updated to be safely used in children using pediatric rhythm libraries [67, 68]. The characteristics of the rhythms in these libraries are similar to the publicly available databases, and are frequently obtained from in-hospital electrophysiology studies [11, 68]. However, when the algorithms are used to diagnose rhythms in OHCA patients the Se/Sp values reported are lower. For instance, a recently described AED algorithm [11] presented Se/Sp of 99.5/98.0% when tested on proprietary rhythm databases, but when tested with OHCA data in the ventilation pauses during 30:2 CPR the Se/Sp decreased to 93.8/95.9%, respectively [69]. Similar results were found for another AED algorithm with OHCA rhythms and short ECG analysis segments of 3-5 s [70]. So further research on VF detection using OHCA data is needed, and the deep learning approach proposed in this study is a contribution in that direction.

Two recent papers have introduced deep learning architectures based on 1D-CNNs for the detection of shockable arrhythmia [39, 48]. Both studies are based on data from public ECG repositories, in particular the malignant ventricular arrhythmia were obtained from the CUDB and VFDB databases. As shown by our results (see Table 3) the detection of shockable arrhythmia is easier on data from public repositories than in OHCA data, a result that had already been proved using classical machine learning algorithms [27]. OHCA data is recorded using defibrillators in a pre-hospital setting, and by the time the emergency services arrive the condition of the myocardium may have considerably deteriorated. This implies that the nonshockable rhythms recorded during OHCA most frequently correspond to pulseless patients, have irregular rates, and aberrant QRS complexes in patients with conduction problems [71]. VF is also recorded minutes after its onset, and by that time VF amplitude, fibrillation frequency, and waveform complexity have normally deteriorated [72, 73]. Furthermore, in OHCA there are frequent rhythm transitions from shockable to non-shockable rhythms as a result of the therapies applied by the emergency services [74], so borderline rhythms are more frequent. Therefore, although using ECGs from public databases is a standard practice and provides a good benchmark for the development and comparative assessment of VF detection methods [23, 24], it is desirable to use OHCA data to develop and test VF-detection methods. Our results show that the BAC were 1-3 points lower for the OHCA data than for the data from public ECG repositories, depending on the classifier (SVM or deep learner) and the segment length used for classification.

The design of our deep learning architecture combines 1D-CNN blocks with an LSTM network. The solution considerably improves the performance of some previous deep learning approaches [34, 56], of which some were specifically introduced to detect lethal ventricular arrhythmia [39]. Those architectures were based only on convolutional networks, and did not include the LSTM stage introduced in this study to integrate the temporal relations in the ECG. The benefits of adding an LSTM block are larger for longer segment lengths (see Fig 3) because then the LSTM network efficiently captures the temporal relations of the arrhythmia. An advantage of using an LSTM is that the network is able to learn the long term temporal relations of the ECG. LSTMs can capture different time scales and can capture subtle ECG nuances for arrhythmia classification [75, 76]. As shown in Table 3, the addition of an LSTM block improves the VF-detection accuracy of the deep learning architectures based only on CNN blocks. The increase in BAC over the best CNN only architecture is 0.5-points for public data, but over 2-points for the more challenging OHCA data. Acharya et al [39] reported an Se and Sp of 91.0% and 95.3% (BAC, 93.3%) for the public datasets in the first study that introduced CNN networks for the detection of lethal ventricular arrhythmia. Our implementation of their network yielded a much higher BAC of 98.2% on the public datasets, but we used a 4-s segment and cleaned the data of artifacts, intervals with loss of signal, asystole, and intermediate rhythms. These differences increase the BAC since classification is easier on longer segment lengths (see Fig 3), and noisy data is a confounding factor for the design of the classifiers. The recent contribution by Nguyen et al [48] used 8-s ECG segments and data cleaning, and reported Se and Sp of 97.1% and 99.4% (BAC, 98.3%). Their results are therefore similar, when compared on the same conditions, to the values we obtained for the model proposed by Acharya et al. In any case, the addition of the LSTM block results in at least 1-point increase in BAC for the public datasets over those two architectures. It is important to emphasize that in practice an AED instructs the rescuer to stop CPR and avoid movements to analyze the rhythm, so the ECG observed by the device is free of artifacts. In the event of a low quality signal or loss of signal the device would not analyze the ECG. VF detection algorithms should therefore be tested under these conditions, as recommended by the AHA [5].

Most studies introducing or comparing algorithms for the detection of malignant ventricular arrhythmia use 4- or 8-s analysis segments [23, 24, 27–29, 31, 32, 48]. The duration of the interruptions in CPR therapy associated to an AED analysis ranges from 5.2 − 28.4 s depending on the AED model [77]. An important factor in this time is the ECG segment length needed by VF-detection algorithms to classify the rhythm as shockable or non-shockable. Consequently, shortening the analysis segment would shorten hands-off intervals (without CPR) during AED use, and shorter hands-off intervals increase the OHCA survival rates [78]. Moreover, reducing the analysis segment to around 3-s would enable the continuous monitoring of the rhythm during 30:2 CPR (30 compressions and 2 ventilations) because the rhythm could be analyzed during the ventilation pauses [69]. Our results for the OHCA data show that AHA requirements for Se and Sp are met for analysis segments of at least 3-s. When we tested a 2-s analysis segment the Sp on the OHCA data fell to 93.6%, below the 95% AHA target, and there was an overall decrease in BAC from 98.9% for 8-s segments to 95.2% for 2-s segments. In contrast, on the public data the BAC decreased only from 99.6% for 8-s segments, to 98.1% for 2-s segments, and the Se and Sp were above 97.5% for all the tested segment lengths (see Figs 3 and 4). These results suggest that capturing the characteristics of OHCA arrhythmia is difficult using short segment lengths, specially for nonshockable rhythms in which heart rates are frequently very slow and QRS complexes may be very wide due to aberrant ventricular conduction. It is therefore important to use OHCA data to explore the limits of accurate VF detection in cardiac arrest patients. In any case, our deep learning architecture was capable of an AHA compliant diagnosis for segment lengths as short as 3-s, shorter than the typical AED analysis duration which is above 5-s [77].

Finally, we bench-marked the results of our deep learning architecture against a state of the art machine learning algorithm using SVM and a large set of well known hand crafted VF-detection features. Our deep learning architecture outperformed the classical machine learning approach on both the public and the OHCA datasets and for all segment lengths, a trend observed in several other ECG classification problems [34, 37, 40, 41]. The increase in BAC was marginal for the public dataset, for instance for 4-s segments the BAC was only 0.3-points higher. However, for the OHCA there was a substantial increase in BAC which was 1.5 to 3-points larger depending on the segment length. Our deep learning architecture learned a set of 20 features output by the LSTM network. These features showed better separability, and performed better than the classical hand-crafted features when fed to the SVM classifier. Deep learning approaches can therefore be conceived and exploited to obtain improved ECG classification features, as proposed by Nguyen et al [48].

Deep learning solutions have proven effective and accurate for VF detection during OHCA. In the future several other clinical decision support algorithms during OHCA could benefit from using deep learning solutions. Some areas of current research include the prediction of shock success [79, 80], the analysis of the ECG during manual and mechanical chest compressions [81, 82], multiclass rhythm classification [30], or the detection of pulse [83]. In fact some deep learning solutions have been recently proposed for instance for the detection of pulse [84]. Moreover, given the difficulties in gathering annotated OHCA data it would be of great interest to investigate the possibility of using these initial approaches in solutions with improved data efficiency, such as few shot learning solutions.

## Conclusion

We have designed a mixed CNN and LSTM deep learning architecture for the detection of lethal ventricular arrhythmia. Our results show that this network outperforms the classical machine learning algorithms and the deep learning architectures based only CNN layers that had been proposed to date for this task. Furthermore, our results are tested on public repositories of ECG data but also on OHCA data which is harder to classify. Our architecture enabled an AHA compliant shock/no-shock decision for analysis segments as short as 3-s. Finally, the computational demands of our model fit in the low-end hardware of an AED, and implementing it on an AED would be low cost since it only involves a software change to the device.

## Supporting information

S1 Fig Performance of all deep learners for different ECG segment lengths. In the figure CNN_1_ refers to Kiranaz et al [34], CNN_2_ to Zubair et al [56], and CNN_3_ to Acharya et al [39]. The networks CNN_2-3_ perform worse for longer segment length, they are more complex and need to adjust more weights (see Table 2) and perform better with more instances in the dataset. The advantages of using an LSTM block diminish for shorter segment lengths, as the LSTM is not able to capture the long temporal relations in the arrhythmia.
(TIFF)
