# Supplementary figures and images for: Mixed convolutional and long short-term memory network for the detection of lethal ventricular arrhythmia
Source: PLoS One. 2019 May 20;14(5):e0216756. doi: 10.1371/journal.pone.0216756 (PMC6527215; doi:10.1371/journal.pone.0216756)

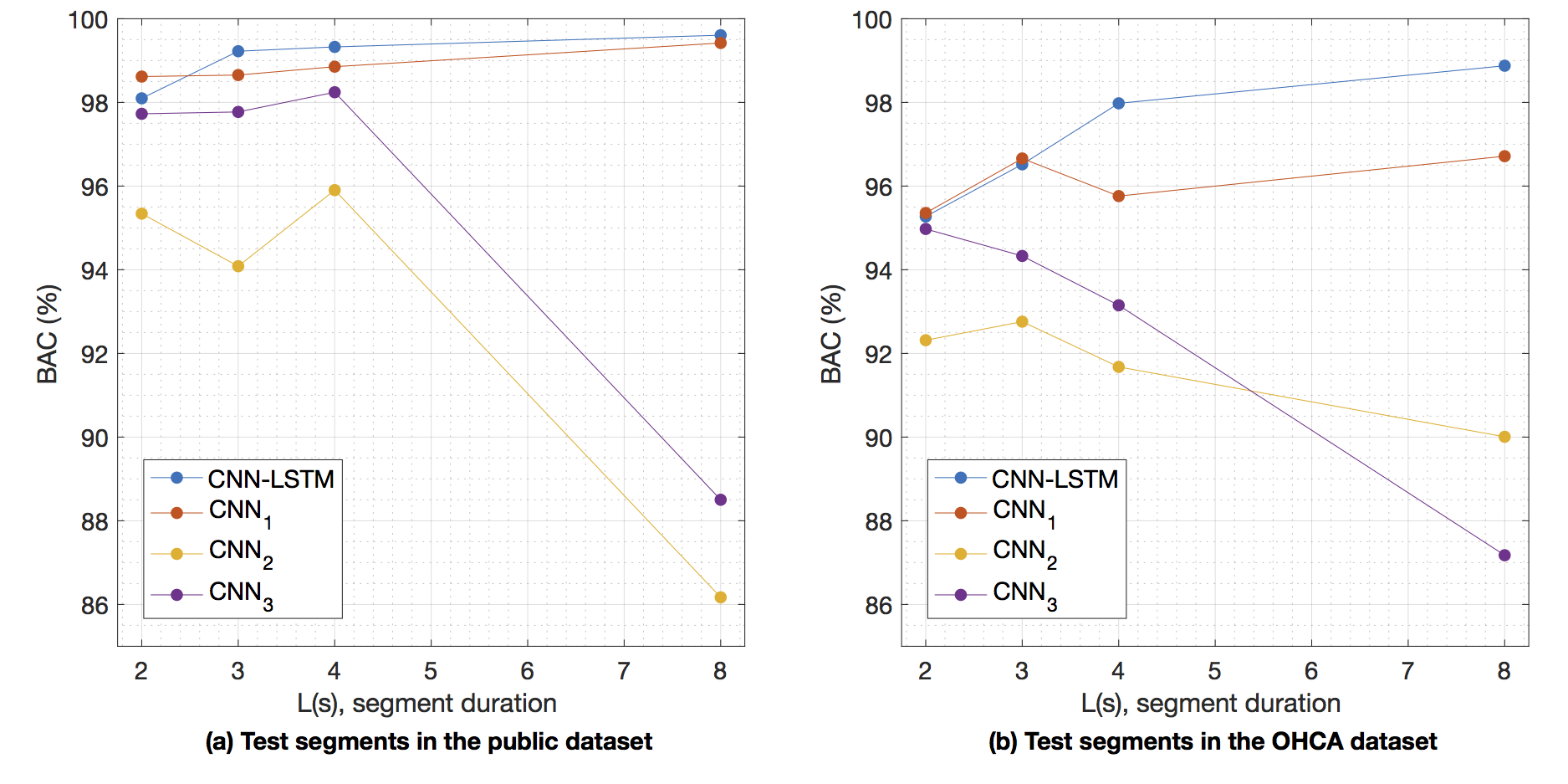

Supplement: S1 Fig — In the figure CNN1 refers to Kiranaz et al [34], CNN2 to Zubair et al [56], and CNN3 to Acharya et al [39]. The networks CNN2-3 perform worse for longer segment length, they are more complex and need to adjust more weights (see Table 2) and perform better with more instances in the dataset. The advantages of using an LSTM block diminish for shorter segment lengths, as the LSTM is not able to capture the long temporal relations in the arrhythmia. (TIFF) [file pone.0216756.s001.tiff]
